# Knowledge, willingness, and self-efficacy in CPR: A two-phase study of prevalence and preliminary peer-led workshop in a Malaysian tertiary institution

**DOI:** 10.1371/journal.pone.0360510

**Published:** 2026-10-12

**Authors:** Siew-Keah Lee, Lei Jie Hoo, Rachel Zoe Koh Raj, Irvin Ken Lym Yeo, Yu Wen Beh, Rosidah Ibrahim, Kai Bin Liew, Yik-Ling Chew, Eugenie Sin Sing Tan, Ang-Lim Chua

**Affiliations:** 1 M. Kandiah Faculty of Medicine and Health Sciences, Universiti Tunku Abdul Rahman, Selangor, Malaysia; 2 Faculty of Pharmacy, University of Cyberjaya, Selangor, Malaysia; 3 Faculty of Pharmaceutical Sciences, UCSI University, Negeri Sembilan, Malaysia; 4 School of Healthy Aging, Medical Aesthetics and Regenerative Medicine, Faculty of Medicine and Health Sciences, UCSI University, Kuala Lumpur, Malaysia; 5 Faculty of Medicine, Universiti Teknologi MARA, Selangor, Malaysia; Universiti Sains Malaysia, MALAYSIA

## Abstract

**Background:**

Out-of-hospital cardiac arrest remains a major public health concern, and early cardiopulmonary resuscitation (CPR) by bystanders is crucial for improving survival outcomes. University students represent an important target population for CPR education because they are socially active, highly accessible, and capable of disseminating lifesaving knowledge within the wider community. However, inadequate CPR knowledge, low willingness to perform CPR, and poor self-efficacy remain common among university students in Malaysia.

**Objectives:**

This two-phase study aims to determine the prevalence of self-reported CPR-related knowledge, willingness, and self-efficacy among students in a Malaysian tertiary institution (Phase 1) and to explore the preliminary changes in these outcomes following a peer-led CPR workshop (Phase 2).

**Methods:**

In Phase 1, a cross-sectional survey was conducted at a tertiary institute in Selangor, Malaysia using a validated questionnaire on knowledge, attitudes, behavioral intention, and self-efficacy (KABSE) (N = 431). In Phase 2, a two-hour peer-led workshop, delivered by medical students, targeted students without CPR training in the past two years, and pre–post KABSE changes were measured (N = 33).

**Results:**

Phase 1 baseline findings revealed CPR knowledge and self-efficacy levels were relatively lower than attitudes and willingness to perform CPR. Females showed higher knowledge and attitudes, while males reported greater self-efficacy. Older and married participants demonstrated better attitudes, and those with a healthcare-related background had higher knowledge, attitudes, and self-efficacy, though similar behavioral intention. Higher scores were also linked to AED awareness, AED usage capability, and recent CPR training. Statistical analysis revealed significant associations among the KABSE domains. Following the peer-led workshop, the participants showed significant gains in self-reported knowledge (p < 0.001), attitude (p < 0.001), behavioral intention (p < 0.01), and self-efficacy (p < 0.001).

**Conclusion:**

Although participants displayed positive attitudes toward CPR, baseline knowledge and self-efficacy were limited. The peer-led workshop demonstrated feasibility as a proof-of-concept intervention, generating significant short-term gains in self-reported knowledge and confidence.

## Introduction

Out-of-hospital cardiac arrest (OHCA) is a time-critical emergency with high mortality, particularly when immediate intervention is lacking. Cause can be cardiac (e.g., acute myocardial infarction, malignant arrhythmias, cardiomyopathies, and severe heart failure) and non-cardiac (e.g., airway obstruction, drowning, trauma, electrocution, drug overdose, and massive hemorrhage) Unlike in-hospital cardiac arrests, OHCA outcomes rely heavily on rapid recognition, prompt activation of emergency medical services (EMS), and timely bystander cardiopulmonary resuscitation (CPR) [1–5]. Survival following cardiac arrest declines by approximately 7–10% for every minute that ventricular arrhythmia remains untreated [6]. This makes timely bystander CPR the single most effective intervention in bridging the gap until EMS arrives.

Globally, bystander CPR rates vary dramatically depending on local emergency systems and public education initiatives, ranging from 1.3 to 72% [7]. High-income Western nations often report layperson CPR rates exceeding 50–70% [8], whereas regional Asian counterparts such as Singapore (~59%) and South Korea (~47%) have achieved substantial gains through nationwide public-access training and automated dispatch initiatives [9]. In Malaysia, however, bystander CPR engagement remains relatively low at approximately 38%, while automated external defibrillator (AED) utilization is under 1.5% [10]. Coupled with mean EMS response times of roughly 12 minutes, which exceeding the recommended 8 minutes window [10,11], this low bystander intervention rate creates a critical pre-hospital gap that severely undermines OHCA survival outcomes nationally [12].

Determinants of willingness to perform CPR/AED include demographics, prior training, attitudes, self-efficacy, and legal protection. Positive factors are younger age, male gender, higher education, recent CPR/AED training, repeated exposure, and confidence [2–5]. Barriers include fear of litigation, harming the victim, and misconceptions that only medical professionals should perform CPR [3,4]. While training improves knowledge and willingness [13], participation is often hindered by cost, travel, and time commitments [14].

In Malaysia, current public health initiatives related to CPR have predominantly targeted medical and healthcare professionals, with limited availability of training opportunities for the public. In this context, colleges or universities represent an untapped yet promising platform for CPR education. As institutions composed of individuals from diverse academic, cultural, and socioeconomic backgrounds, universities mirror the broader Malaysian society. More importantly, they provide a structured and supportive environment that is ideal for implementing systematic CPR training and awareness initiatives. Students, lecturers, and staff are often in the age group that is both likely to witness and respond to OHCA incidents, whether in university, at home, or in public settings. The most accessible and practical time to equip individuals with life-saving CPR skills is during their university years, a period marked by openness to learning and exposure to health campaigns and co-curricular development. Furthermore, once students graduate, they disperse into various sectors and locations across Malaysia and globally, making them powerful carriers of CPR knowledge and readiness. Despite the potential of this setting, the wider university community including students from non-health faculties remains relatively neglected in CPR-related studies. These individuals are equally likely to be present during cardiac emergencies but may lack the basic competencies or willingness to intervene [2,15]. This oversight presents a critical gap in both academic research and national preparedness for OHCA.

Variables such as academic discipline, age, gender, prior CPR exposure, and awareness of emergency protocols may influence a person’s preparedness to act. These associations remain underexplored, and addressing this gap is essential for developing targeted, inclusive and peer-led CPR workshops across campus communities. Therefore, Phase 1 of this study aimed to examine the levels of CPR-related knowledge, willingness, and self-efficacy among the university community and to explore the relationship between these levels and various sociodemographic characteristics. Phase 2 explored a brief peer-led CPR workshop as a proof-of-concept educational approach, focusing on preliminary changes in CPR knowledge, willingness, and self-efficacy following the training. Together, the two phases provide an initial understanding of CPR preparedness within the university community and the potential of peer-led training to address identified gaps in CPR knowledge, willingness to perform CPR, and self-efficacy.

## Methods

### Study design and ethical considerations

This study was conducted in strict accordance with the ethical standards set forth by the institutional ethics committee to ensure the protection, rights, and confidentiality of all participants. Ethical approval was obtained from the Universiti Tunku Abdul Rahman Scientific and Ethical Review Committee (Approval Number: U/SERC/78–546/2025). The subject recruitment period was from 27/06/2025 to 31/08/2025. Written informed consent was obtained from all participants prior to their enrolment in the study.

### Survey instrument and participant recruitment

Both Phase I and II employed a self-administered questionnaire previously validated in the literature. The instrument comprised five main sections assessing knowledge, attitude, behavioral intention, and self-efficacy, demonstrating high content validity (content validity index = 0.95) and high internal consistency (Cronbach’s alpha = 0.92) [16].

Participants for the Pilot Study, Phase 1, and Phase 2 were recruited using convenience sampling through an e-mail announcement disseminated via the university’s Mail-Master system. Participation was voluntary, and eligible individuals self-selected to participate. participants were eligible if they were aged 18 years or above, able to read and understand English, and able to provide informed consent.

### Pilot study

Prior to commencement of the main study, a pilot study with 30 participants was conducted to re-evaluate the clarity, feasibility, and reliability of the KABSE questionnaire, yielding Cronbach’s alpha coefficients exceeding 0.95 across all domains. Given the small sample size, this pilot was not intended to re-establish full psychometric validity, but ensured the questionnaire was understandable and reliable prior to use in the main study.

### Phase 1 Study: Assessment of KABSE and its association with sociodemographic determinants

Participants who had participated in the Pilot Study were excluded from Phase 1. Recruited participants completed the self-administered KABSE questionnaire to assess their knowledge, attitude, behavioral intention, and self-efficacy toward CPR. Sociodemographic information was also collected for subsequent analysis of the associations between participant characteristics and KABSE scores.

### Phase 2 Study: Pre- and post-workshop assessment

A quasi-experimental intervention design was employed in Phase 2. This design was selected because participants were not randomly assigned; instead, they voluntarily enrolled in the peer-led CPR workshop. Participants who had participated in the Pilot Study and Phase 1 were excluded from Phase 2 to minimize potential testing and carryover effects. Participants were required to have no formal recent CPR training within the preceding two years. Recent CPR training was operationally defined as training received within the preceding two years. This cutoff was selected pragmatically to represent relatively recent CPR exposure, considering that CPR certification commonly has a validity period of two years. Nevertheless, this classification was not intended to imply that CPR-related knowledge, willingness, or self-efficacy ceases to be retained after 2 years, but rather to distinguish relatively recent training exposure from more remote training experience.

All eligible participants completed pre- and post-intervention assessments to evaluate changes in knowledge, attitude, behavioral intention, and self-efficacy toward CPR following the training. The training was facilitated by four peer instructors, all of whom were Year 2 medical students and certified first aiders. Prior to leading the workshop, they successfully completed a refresher Basic Life Support (BLS) Provider course delivered by an emergency medicine specialist and organized by the Selangor Committee on Resuscitation Training (SCORT). All peer instructors achieved passing standards in both theoretical and practical assessments. This specialist-led preparation ensured their competence to deliver high-quality CPR instruction.

Upon arrival, participants completed a pre-workshop questionnaire to collect sociodemographic information and baseline KABSE scores. The workshop began with a theory session covering the chain of survival, BLS steps, and choking response protocols. This was followed by a practical component conducted at two stations: Station 1 (choking management) and Station 2 (CPR training). Participants were divided into two large groups, rotating between stations after 25 minutes.

Within the CPR station, smaller subgroups of four to five participants practiced complete CPR cycles and the recovery position on manikins under close observation. Each participant underwent hands-on CPR training guided by a standardized BLS performance checklist covering seven critical domains: Danger, Response, Shout for Help, Airway, Breathing, Circulation, and Pulse Check. The checklist was used by trainers as an instructional guide to ensure consistent demonstration and practice of the key steps of CPR. Throughout the practical session, participants received immediate, individualized feedback to facilitate timely correction of errors and reinforce proper technique. No written evaluation of participants’ practical performance was conducted or analyzed.

At the choking station, participants were taught and practiced both conscious and unconscious choking management using a choking mannequin and live models. Each participant’s performance was directly observed, and immediate corrective feedback was provided to ensure proper technique and reinforce key safety principles.

After completing both the theory and practical sessions, participants filled out the post-workshop KABSE questionnaire. The entire training lasted approximately two hours and was provided free of charge.

Knowledge was measured through a 12-single best answer questions related to CPR, a correct response was awarded one mark, while incorrect or unanswered questions were was given zero marks, with higher scores indicating greater knowledge.

Attitude, behavioral intention, and self-efficacy toward CPR were measured using a 5-point Likert scale ranging from 1 (strongly disagree/strongly unwilling/not confident at all) to 5 (strongly agree/strongly willing/extremely confident). Item scores were summed up to produce domain totals, with higher scores indicating more positive attitudes, stronger behavioral intention, and greater self-efficacy. The total scores for Knowledge, Attitude, Behavior and Self-Efficacy domains were transformed into percentage scores to permit comparisons across domains with differing item counts.

### Statistical analysis

Data were analyzed using IBM SPSS Statistics Version 30.0. The distribution of continuous variables was assessed using the Kolmogorov–Smirnov test. As p-values were less than 0.05, the data were considered non-normally distributed and are presented as medians with interquartile ranges (IQRs) and further analyzed with non-parametric tests. Differences in KABSE scores across sociodemographic groups were examined using the Mann-Whitney U test for comparisons between two groups and the Kruskal-Wallis test for comparisons among more than two groups, followed by Bonferroni-adjusted pairwise comparisons where appropriate. Spearman’s rank correlation was applied to assess relationships between KABSE components. While The Wilcoxon signed-rank test was used to compare pre- and post-workshop KABSE scores. Subsequently, Cohen’s effect size was determined and interpreted as small (0.10–0.29), moderate (0.30–0.49), and large (≥0.50) [17].

## Results

### Phase 1 Study: Assessment of KABSE and its association with sociodemographic determinants

A total of 431 participants were enrolled into Phase 1 study. Median (IQR) baseline scores were as follows: knowledge 50.00 (33–67), attitude 83.00 (73–95), behavioral intention 75.00 (63–88), and self-efficacy 31.00 (19–50) (Table 1). Individual item for each KABSE domain is reported in Supplementary Data (S1–S4 Tables).

**Table 1:** Median scores in knowledge on CPR, attitude towards CPR, behavioral intention in performing CPR, and self-efficacy in performing CPR.

Variables	Median (IQR)
Knowledge on CPR	50.00 (33–67)
Attitude towards CPR	83.00 (73–95)
Behavioral intention to perform CPR	75.00 (63–88)
Self-efficacy to perform CPR	31.00 (19–50)

The main barriers to training included lack of knowledge of where to learn (30.2%) and time constraints (29.9%) (Table 2). The most frequently cited reason for unwillingness to perform CPR was concern about causing harm due to lack of hands-on experience, reported by 316 participants (73.3%). Fear of legal repercussions was the second most common reason (46.6%), followed by discomfort in performing mouth-to-mouth resuscitation on a stranger (38.1%). Other barriers included fear of contracting infections or diseases when assisting strangers (30.2%) and concern about being judged for performing CPR incorrectly (29%) (Table 3).

**Table 2:** Reasons for not attending CPR training.

Item	n (%)
Want to learn but don’t know where to learn	130 (30.2)
Too busy with work and studies; no time to learn	129 (29.9)
Not relevant to me; don’t want to participate	39 (9)
Don’t think I’ll ever need it, so don’t want to learn	20 (4.6)
Physical condition doesn’t allow	14 (3.2)
Others	21 (4.9)

**Table 3:** Reasons for unwillingness to perform CPR.

Item	n (%)
I worry that my lack of hands-on experience might cause harm to the person	316 (73.3)
I am afraid of being sued or held legally responsible for any adverse outcomes	201 (46.6)
I feel uncomfortable performing mouth-to-mouth resuscitation on someone I do not know	164 (38.1)
I am hesitant to perform CPR on a stranger due to fear of contracting infections or diseases	130 (30.2)
I am concerned about being judged by others if I perform CPR incorrectly	125 (29)
Others	7 (1.6)

Females demonstrated higher levels of knowledge and more positive attitudes compared to males; however, males reported greater self-efficacy. Older and married participants showed better attitudes toward CPR. Participants with an academic healthcare-related background (Medicine, Chinese Medicine, Physiotherapy or Nursing) consistently exhibited higher knowledge, attitudes, and self-efficacy, although their behavioral intention to perform CPR was comparable to those without such an academic background. Higher knowledge, attitude, and self-efficacy scores were also associated with AED awareness, AED usage capability, and recent CPR training (p < 0.001) (Table 4).

**Table 4:** Significance difference between KABSE and sociodemographic factors and CPR training exposure.

Variables	n (%)	Knowledge		Attitude		Behavioral Intention		Self-efficacy	
		Median [IQR]	*p*-value	Median[IQR]	*p*-value	Median[IQR]	*p*-value	Median[IQR]	*p*-value
**Gender**									
Male	182 (42.2)	50.00[33.33-66.67]	.027*	80.00[70.00-90.63]	.006**	75.00[60.94-93.75]	NS	37.50[25.00-50.00]	.010*
Female	249 (57.8)	50.00^[33.33-66.67]		82.50[75.00-95.00]		75.00[62.50-87.50]		25.00[18.75-50.00]	
**Age**									
18-25	341 (79.1)	50.00 [33.33-66.67]	NS	80.00 [71.25-92.50]	.002**^a^	75.00 [62.50-87.50]	NS	31.25[18.75-50.00]	NS
26-40	49 (11.4)	50.00 [33.33-75.00]		85.00[77.50-97.50]		75.00 [71.88-93.75]		31.25[18.75-50.00]	
41 and above	41 (9.5)	41.67 [20.83-58.33]		87.50[80.00-96.25]		75.00[62.50-100.00]		37.50[21.88-56.25]	
**Marital Status**									
Single	372 (86.3)	50.00[33.33-66.67]	NS	80.00 [72.50-92.50]	.004**	75.00 [62.50-87.50]	NS	31.25 [18.75-50.00]	NS
Married	59 (13.7)	50.00[25.00-66.67]		87.50 [77.50-97.50]		75.00[62.50-100.00]		43.75 [25.00-56.25]	
**Healthcare Background**									
Healthcare-related	86 (20)	58.33[41.67-83.33]	<.001***	90.00 [72.50-100.00]	.003**	75.00 [62.50-93.75]	NS	50 [25, 68.75]	<.001***
Non-healthcare-related	345 (80)	50.00 [33.33-66.67]		80.00 [72.50-90.00]		75.00 [62.50-87.50]		25.00 [18.75-50.00]	
**Have you heard of Cardiopulmonary Resuscitation (CPR)?**									
Yes	409 (94.9)	50.00[33.33-66.67]	<.001***	82.50[72.50-95.00]	0.014*	75.00[62.50-87.50]	NS	31.25[18.75-50.00]	NS
No	22 (5.1)	20.83[8.33-43.75]		73.75[63.75-85.00]		75.00[60.94-95.31]		25.00[9.38-50.00]	
**Have you ever encountered a situation where someone needed CPR outside of a hospital?**									
Yes	69 (16)	50.00[33.33-66.67]	NS	80.00[71.25-93.75]	NS	75.00[62.50-93.75]	NS	31.25[25.00-50.00]	NS
No	362 (84)	50.00[33.33-66.67]		82.50[72.50-95.00]		75.00[62.50-87.50]		31.25[18.75-50.00]	
**Have you performed CPR on someone else?**									
Yes	19 (4.4)	58.33[33.33-66.67]	NS	77.50[70.00-95.00]	NS	100.00[75.00-100.00]	.021*	50.00[31.25-62.50]	.023*
No	412 (95.6)	50.00[33.33-66.67]		82.50[72.50-94.38]		75.00[62.50-87.50]		31.25[18.75-50.00]	
**Have you heard of Automated External Defibrillators (AED)?**									
Yes	249 (57.8)	58.33[41.67-75.00]	<.001***	85.00[75.00-95.00]	<.001***	75.00[62.50-93.75]	<.001***	37.50[25.00-56.25]	<.001***
No	182 (42.2)	41.67[25.00-52.08]		77.50[70.00-88.13]		75.00[62.50-87.50]		25.00[12.50-50.00]	
**Are you capable of using AED?**									
Yes	68 (15.8)	75.00[58.33-83.33]	<.001***	95.00[77.50-100.00]	<.001***	81.25[68.75-100.00]	.005**	62.50[50.00-75.00]	<.001***
No	363 (84.2)	50.00[33.33-58.33]		80.00[72.50-90.00]		75.00[62.50-87.50]		25.00[18.75-50.00]	
**Are you aware that Automated External Defibrillators (AEDs) are available in UTAR?**									
Yes	130 (30.2)	62.50[41.67-77.08]	<.001***	87.50[77.50-97.50]	<.001***	75.00[62.50-93.75]	0.045*	50.00[25.00-68.75]	<.001***
No	301 (69.8)	41.67[33.33-58.33]		80.00[71.25-90.00]		75.00[62.50-87.50]		25.00[12.50-50.00]	
**Have you ever seen an AED device in any public facility?**									
Yes	203 (47.1)	58.33[41.67-75.00]	<.001***	85.00[75.00-95.00]	.003**	75.00[62.50-93.75]	.003**	43.75[25.00-56.25]	<.001***
No	228 (52.9)	41.67[33.33-58.33]		80.00[70.00-90.00]		75.00[62.50-87.50]		25.00[12.50-50.00]	
**Have you attended CPR training?**									
Yes, in the past 2 years	56 (13)	75.00[66.67-83.33]	<.001*** ^a^	95.00 [83.13-100.00]	<.001*** ^a^	75.00 [62.50-93.75]	.023* ^a^	62.50 [50.00-75.00]	<.001*** ^a^
Yes, between 2 to 5 years ago	27 (6.3)	58.33 [41.67-70.00]		90.00 [80.00-100.00]		87.50 [75.00-100.00]		50.00 [25.00-75.00]	
Yes, more than 5 years	68 (15.8)	50.00[33.33-66.67]		82.50 [75.00-94.38]		75.00 [62.50-93.75]		37.00 [18.75-50.00]	
No	280 (65)	41.67[33.33-58.33]		80.00 [70.00-87.50]		75.00 [62.50-87.50]		25.00[12.50-43.75]	
**Are you a certified first aider?**									
Yes, I am an active first aider	28 (6.5)	79.17[58.33-91.67]	<.001***^b^	97.50[83.70-100.00]	<.001*** ^b^	87.50 [70.31-100.00]	.033*^b^	75.00 [57.81-98.44]	<.001*** ^b^
Yes, I was a previous first aider.	42 (9.7)	58.33[41.67-75.00]		88.00[71.88-100.00]		75.00 [62.50-100.00]		50.00 [25.00-68.75]	
No	361 (83.8)	50.00[33.33-66.67]		80.00 [72.50-90.00]		75.00 [62.50-87.50]		25.00[18.75-50.00]	

^Female has higher mean value; *p < 0.05, **p < 0.01, ***p < 0.001, NS: non-statistically significance. ^a^ in the past 2 years compared to counterparts; ^b^ active first aider compared to counterparts

Significant positive correlations were observed between knowledge and attitude (r = 0.297, p < 0.001), knowledge and self-efficacy (r = 0.213, p < 0.001), and attitude and behavioral intention (r = 0.477, p < 0.001).

### Phase 2 study: Pre- and post-workshop assessment

A total of 33 participants were enrolled into Phase 2 study. As shown in Table 5, all KABSE domains improved significantly after the workshop. Knowledge increased from a median of 58.33 to 91.67 with a large effect size (r = 0.853). Attitude improved with a moderate effect size (r = 0.578), while behavioral intention showed a moderate effect (r = 0.476). Self-efficacy increased from 50.00 to 75.00 with a large effect size (r = 0.794). All changes were statistically significant, the effect sizes demonstrate that the magnitude of improvement, particularly for knowledge and self-efficacy was practically meaningful (Table 5).

**Table 5:** Median Scores in knowledge, attitude, behavioral intention, and self-efficacy towards CPR pre and post the workshop.

Variables	Pre-scores Median (Q1-Q3)	Post-scores Median (Q1-Q3)	Z-value	*p*-value	Effect size (r)	Interpretation-on
Knowledge	58.33 (42–79)	91.67 (83–100)	4.902	<0.001	0.853	Large
Attitude	90.00 (78–100)	100.00 (90–100)	3.317	<0.001	0.578	Large
Behavioral intention	75.00 (63–94)	87.50 (75–100)	2.735	<0.05	0.476	Moderate
Self-efficacy	50.00 (31–56)	75.00 (63–81)	4.560	<0.001	0.794	Large

Cohen’s effect size (r) is interpreted as small (0.10–0.29), moderate (0.30–0.49), and large (≥0.50).

## Discussion

This study found that despite generally positive attitudes and behavioral intentions towards CPR, participants demonstrated lower knowledge and self-efficacy. The Phase 1 results indicate that while participants value CPR, they lack sufficient knowledge and confidence to perform it. This discrepancy highlights the need for targeted educational interventions combining theoretical knowledge and hands-on practice to strengthen CPR knowledge, willingness, and self-efficacy. Accordingly, the Phase 2 peer-led workshop was designed as a proof-of-concept evaluation to explore the feasibility of this educational approach and its preliminary effects on the study’s prespecified outcomes. The primary outcomes were CPR knowledge, willingness to perform CPR, and self-efficacy; the intervention was not designed to assess or establish CPR competency. Thus, the practical component served to reinforce learning and provide participants with an opportunity to practice key CPR steps, rather than to generate an objective measure of practical competency. Further studies may build upon these findings by incorporating formal competency assessments to determine whether improvements in knowledge, willingness, and self-efficacy translate into improved CPR performance.

Female participants may be more likely to perceive CPR as an important life-saving skill, leading to higher knowledge acquisition and greater willingness to perform the procedure in an emergency. Males, on the other hand, had higher self-efficacy scores, indicating that they felt more confident in their ability to perform CPR. This gender difference in self-efficacy could be also influenced by societal perceptions of male competence in emergency situations, where males may feel more physically capable of performing CPR despite lacking sufficient training [18–20]. These findings highlight the need for gender-sensitive training strategies that both reinforce the positive attitudes and knowledge acquisition observed among females while ensuring that male participants develop competence that matches their confidence.

Furthermore, individuals with a healthcare-related background or prior CPR exposure and training demonstrated significantly higher levels of KABSE. These differences highlight the importance of tailoring CPR training programs to address the specific needs of various demographic groups. For example, individuals from non-healthcare background may benefit from more basic CPR education, while those with healthcare foundation may require advanced training or refresher courses to ensure their knowledge and skills remain up to date.

Multiple studies conducted across different Malaysian populations have consistently reported unsatisfactory levels of CPR knowledge and self-efficacy, with evidence demonstrating a significant statistical association between the two [21–24]. Undoubtedly, these shortcomings can be addressed through structured CPR training. Although CPR training is widely available in Malaysia through both governmental and non-governmental agencies, participation remains low, which may contribute to the persistently inadequate levels of CPR knowledge and self-efficacy among the local community [12]. Addressing this gap requires a multifaceted approach that reduces financial barriers, increases accessibility, and accommodates time constraints [4,19,25]. Providing subsidized or free training, supported by institutional or corporate initiatives, could alleviate cost-related challenges. Expanding training delivery to community centers, universities, and workplaces, as well as adopting blended models that combine online learning with short, practical workshops, may further enhance accessibility. Furthermore, incorporating CPR modules into academic curricula or professional training requirements would normalize participation and promote sustainability. In addition, public awareness campaigns and peer-led training models can increase motivation and engagement, making CPR education more acceptable and scalable across diverse populations.

Beyond logistical and accessibility challenges, this study highlighted several psychological barriers that hinder participants from performing CPR, including fear of legal repercussions, concerns about causing harm, and apprehension regarding infection risk. Addressing these perceptions is critical in future educational initiatives. Evidence suggests that incorporating the experiences of actual responders can help develop more contextualized and relatable training, particularly by addressing the emotional implications of performing CPR on a family member, dispelling misconceptions about harm, clarifying issues related to training and litigation, and acknowledging the potential psychological impact that may follow resuscitation attempts [4,25,26].

The post-intervention findings indicate that the workshop produced meaningful improvements across all assessed domains, with the relative magnitudes of change offering important insights into its impact. While the absolute numerical values of the effect sizes should be interpreted cautiously, their relative magnitudes provide meaningful insight into which domains benefited most. Knowledge and self-efficacy showed the largest relative gains (r = 0.853 and r = 0.794 respectively), reflecting the workshop’s stronger influence on cognitive understanding and confidence. Attitude showed an improvement (r = 0.578), while behavioral intention exhibited the smallest, though still meaningful change. This pattern is not unexpected, as shifts in motivation and behavioral commitment often require repeated reinforcement or real-world exposure rather than a single session. Overall, the relative distribution of effect sizes indicates that the workshop was especially impactful in improving what participants *know* and how capable they *feel*, while producing more modest but positive changes in their willingness to act, highlighting both the strengths of the intervention and areas that may benefit from additional or repeated training.

These outcomes align with existing evidence indicating that peer-led training in BLS can be useful in enhancing both knowledge and motivational aspects of CPR readiness, and may be as good as professional training [27]. The peer-led set-up likely contributed importantly to this success by creating a supportive learning environment, reducing psychological barriers, and facilitating active engagement. This advantage echoes findings that peer-instructed students experience higher comfort and involvement during training sessions [27,28]. The significant improvement in self-efficacy is particularly meaningful, given its established role as a predictor of actual CPR performance in emergency situations. The peer-led approach also presents a scalable, resource-efficient model. Training facilitated by peers requires fewer professional trainers while maintaining comparable outcomes. Participant feedback via interview reinforced these quantitative outcomes, attendees in this present study valued the hands-on, peer-led format for its clarity, relatability, and anxiety-reducing qualities.

Beyond individual gains, the workshop generated broader institutional benefits by fostering a health-literate and emergency-ready campus culture, consistent with the university’s aim to produce socially responsible graduates. By positioning CPR as a civic responsibility rather than an optional skill, the initiative aligns with public health priorities and empowers the community to respond more effectively to OHCA. To sustain and scale this initiative, peer-led CPR training could be integrated into the mandatory university curriculum, supported by periodic refresher or booster sessions throughout students’ university years. Such reinforcement may help maintain CPR knowledge and self-efficacy and reduce the potential decline in preparedness associated with increasingly remote training exposure. In addition, establishing a “Training of Trainers” (TOT) model to develop successive cohorts of competent peer instructors can support long-term institutionalization, ensuring a self-perpetuating system of CPR readiness and community resilience.

In conclusion, despite generally positive attitudes towards CPR, participants demonstrated limited knowledge and self-efficacy, highlighting a gap between their willingness to engage in CPR and their preparedness to perform it. The Phase 2 peer-led workshop provided proof-of-concept preliminary evidence that such an intervention is feasible and may be associated with improvements in self-reported knowledge and confidence. However, given the single-group pre-post design without a control group, these preliminary findings should not be interpreted as evidence of intervention effectiveness. Further controlled studies incorporating objective CPR skill assessments and longer-term follow-up are warranted to determine whether peer-led training can improve CPR performance and sustain these gains over time.

### Study limitations and future studies

Several limitations should be considered when interpreting the findings of this study. First, Phase 1 relied on convenience sampling at a single institution, which limits the generalizability of the baseline findings across other universities, geographic regions, or non-academic populations. Second, Phase 2 employed a single-arm, pre-post design without a control group and a modest sample size. Consequently, causal inference is constrained, as observed score improvements may be influenced by confounding factors such as testing memory effects, social desirability, or participant maturation. Voluntary recruitment across both phases also introduces self-selection bias, as individuals with higher baseline health literacy or greater intrinsic interest in CPR may have been more inclined to participate.

Third, while the peer-led workshop incorporated a checklist and real-time feedback, detailed psychomotor skills (e.g., chest compression depth and rate) were not formally evaluated. Therefore, post-workshop improvements reflect perceptual and cognitive gains (KABSE domains) rather than verified clinical or practical CPR competency. Fourth, the study evaluated KABSE outcomes immediately following the intervention without longitudinal follow-up, preventing assessment of the retention and sustainability of changes in knowledge and self-efficacy or potential skill decay over time. Although longer-term follow-up was considered, it was not feasible in this present study because some participants had subsequently graduated or left the university, limiting their availability for follow-up assessment.

Future studies should use multicenter, controlled longitudinal designs with larger and more representative samples, incorporating objective CPR skill assessments alongside KABSE measures to confirm whether self-reported gains translate into sustained, high-quality CPR performance. Research should also examine a continuum of CPR training from university-based peer education and periodic refreshers to workplace- and community-based programs, with employers and community organizations playing a role in maintaining CPR preparedness.

## Supporting information

S1 Table Knowledge of CPR. (DOCX)

S2 Table Attitude towards CPR. (DOCX)

S3 Table Behavioural intention to perform CPR. (DOCX)

S4 Table Self-Efficacy to perform CPR. (DOCX)
