# Supplementary material for: Knowledge, willingness, and self-efficacy in CPR: A two-phase study of prevalence and preliminary peer-led workshop in a Malaysian tertiary institution
Source: PLoS One. 2026 Oct 12;21(10):e0360510. doi: 10.1371/journal.pone.0360510 (PMC13657281; doi:10.1371/journal.pone.0360510)
Supplement: Supplementary file 1 [file pone.0360510.s001.docx]

S1 Table. Knowledge of CPR

| Knowledge of CPR | n  (correct) | Percentage  (%) |
| --- | --- | --- |
| Why should CPR be performed immediately when a person's breathing and heartbeat suddenly stops? | 334 | 77.5 |
| How can you assess whether a patient is breathing? | 307 | 71.2 |
| What are the common signs of cardiac arrest in a patient? | 228 | 52.9 |
| Why is it important to gently tap and call out loudly to the patient during an emergency? | 294 | 68.2 |
| Where is the correct location to perform chest compressions during CPR for adults? | 181 | 42.0 |
| What is the correct technique for performing chest compressions during CPR for adults? | 361 | 83.8 |
| What is the recommended rate of chest compressions per minute during CPR? | 127 | 29.5 |
| What is the appropriate depth for chest compressions during CPR for adults? | 153 | 35.5 |
| What is the correct method for opening a patient’s airway during CPR? | 218 | 50.6 |
| What is the recommended compression-to-ventilation ratio for a single rescuer performing on-site CPR? | 82 | 19.0 |
| What is the correct step-by-step sequence for performing CPR according to current guidelines? | 133 | 30.9 |
| How can you assess whether your resuscitation efforts have been successful? | 173 | 40.1 |
| Why should CPR be performed immediately when a person's breathing and heartbeat suddenly stops | 334 | 77.5 |
