# Supplementary material for: Knowledge, willingness, and self-efficacy in CPR: A two-phase study of prevalence and preliminary peer-led workshop in a Malaysian tertiary institution
Source: PLoS One. 2026 Oct 12;21(10):e0360510. doi: 10.1371/journal.pone.0360510 (PMC13657281; doi:10.1371/journal.pone.0360510)
Supplement: Supplementary file 2 [file pone.0360510.s002.docx]

S2 Table. Attitude towards CPR

| Items | Strongly disagree  *n* (%) | Disagree *n* (%) | Neutral  *n* (%) | Agree  *n* (%) | Strongly agree  *n* (%) |
| --- | --- | --- | --- | --- | --- |
| I believe that learning CPR is necessary. | 0 | 1 (0.2) | 36 (8.4) | 158 (36.7) | 236 (54.8) |
| I believe that chest compressions are a critical life-saving measure for patients in sudden cardiac arrest. | 0 | 1 (0.2) | 20 (4.6) | 155 (36.0) | 255 (59.2) |
| CPR is not solely the responsibility of medical personnel; the public should also learn CPR to assist their families or others in need. | 0 | 5 (1.2) | 58 (13.5) | 164 (38.1) | 204 (47.3) |
| I am interested in acquiring CPR knowledge and skills. | 2 (0.5) | 12 (2.8) | 132 (30.6) | 157 (36.4) | 128 (29.7) |
| I am willing to participate in CPR training programs to enhance my knowledge and skills. | 0 | 15 (3.5) | 151 (35.0) | 145 (33.6) | 120 (27.8) |
| I think CPR should be included in school’s curriculum. | 1 (0.2) | 5 (1.2) | 87 (20.2) | 170 (39.4) | 168 (39.0) |
| I believe that first aid equipment, including AEDs, should be made available in public places such as schools, sport centres and shopping malls. | 0 | 2 (0.5) | 24 (5.6) | 156 (36.2) | 249 (57.8) |
| Schools, communities, and workplaces should organize regular CPR training sessions. | 2 (0.5) | 3 (0.7) | 55 (12.8) | 198 (45.9) | 173 (40.1) |
| I consider it a moral responsibility to assist others in need of help. | 2 (0.5) | 6 (1.4) | 69 (16.0) | 172 (39.9) | 182 (42.2) |
| I believe that providing timely first aid can save someone's life. | 1 (0.2) | 1 (0.2) | 23 (5.3) | 158 (36.7) | 248 (57.5) |
