# Supplementary material for: Knowledge, willingness, and self-efficacy in CPR: A two-phase study of prevalence and preliminary peer-led workshop in a Malaysian tertiary institution
Source: PLoS One. 2026 Oct 12;21(10):e0360510. doi: 10.1371/journal.pone.0360510 (PMC13657281; doi:10.1371/journal.pone.0360510)
Supplement: Supplementary file 3 [file pone.0360510.s003.docx]

S3 Table. Behavioural intention to perform CPR

| Items | Strongly unwilling  *n* (%) | Unwilling *n* (%) | Neutral  *n* (%) | Willing  *n* (%) | Strongly willing  *n* (%) |
| --- | --- | --- | --- | --- | --- |
| If a family member, friend, or acquaintance suddenly stops breathing and their heart stops, would you be willing to perform chest compressions on them? | 0 | 7 (1.6) | 49 (11.4) | 164 (38.1) | 211 (49.0) |
| If a family member, friend, or acquaintance suddenly stops breathing and their heart stops, would you be willing to provide rescue breaths for them? | 2 (0.5) | 8 (1.9) | 61 (14.2) | 168 (39.0) | 192 (44.5) |
| Would you be willing to perform chest compressions on a stranger experiencing sudden cardiac arrest? | 8 (1.9) | 27 (6.3) | 128 (29.7) | 162 (37.6) | 106 (24.6) |
| Would you be willing to provide rescue breaths to a stranger experiencing sudden cardiac arrest? | 11 (2.6) | 48 (11.1) | 147 (34.1) | 135 (31.3) | 90 (20.9) |
