# Supplementary material for: Knowledge, willingness, and self-efficacy in CPR: A two-phase study of prevalence and preliminary peer-led workshop in a Malaysian tertiary institution
Source: PLoS One. 2026 Oct 12;21(10):e0360510. doi: 10.1371/journal.pone.0360510 (PMC13657281; doi:10.1371/journal.pone.0360510)
Supplement: Supplementary file 4 [file pone.0360510.s004.docx]

S4 Table. Self-Efficacy to perform CPR

| Items | Not confident at all  *n* (%) | Not confident  *n* (%) | Neutral  *n* (%) | Confident  *n* (%) | Extremely confident *n* (%) |
| --- | --- | --- | --- | --- | --- |
| If someone collapses suddenly, I am confident in my ability to properly assess their consciousness, heartbeat, and breathing. | 59 (13.7) | 138 (32.0) | 136 (31.6) | 69 (16.0) | 29 (6.7) |
| I am confident that I can perform CPR correctly on a patient with no breathing or heartbeat. | 91 (21.1) | 169 (39.2) | 109 (25.3) | 43 (10.0) | 19 (4.4) |
| I am confident that I can perform artificial respiration correctly on a patient without breathing or a heartbeat. | 98 (22.7) | 158 (36.7) | 112 (26.0) | 44 (10.2) | 19 (4.4) |
| I am confident in my ability to use an automated external defibrillator (AED) correctly. | 114 (26.5) | 138 (32.0) | 105 (24.4) | 54 (12.5) | 20 (4.6) |
